# A New Multiple Hypothesis Tracker Integrated with Detection Processing

**DOI:** 10.3390/s19235278

**Published:** 2019-11-30

**Authors:** Ziwei Wang, Jinping Sun, Qing Li, Guanhua Ding

**Affiliations:** 1 School of Electronics & Information Engineering, Beihang University, Beijing 100191, China; wangziwei@buaa.edu.cn (Z.W.); npeight@buaa.edu.cn (G.D.); 2 Department of Engineering, University of Cambridge, Cambridge CB12PZ, UK; ql289@cam.ac.uk

**Keywords:** multiple hypothesis tracker, adaptive detection threshold, score function, sequential probability ratio test

## Abstract

In extant radar signal processing systems, detection and tracking are carried out independently, and detected measurements are utilized as inputs to the tracking procedure. Therefore, the tracking performance is highly associated with detection accuracy, and this performance may severely degrade when detections include a mass of false alarms and missed-targets errors, especially in dense clutter or closely-spaced trajectories scenarios. To deal with this issue, this paper proposes a novel method for integrating the multiple hypothesis tracker with detection processing. Specifically, the detector acquires an adaptive detection threshold from the output of the multiple hypothesis tracker algorithm, and then the obtained detection threshold is employed to compute the score function and sequential probability ratio test threshold for the data association and track estimation tasks. A comparative analysis of three tracking algorithms in a clutter dense scenario, including the proposed method, the multiple hypothesis tracker, and the global nearest neighbor algorithm, is conducted. Simulation results demonstrate that the proposed multiple hypothesis tracker integrated with detection processing method outperforms both the standard multiple hypothesis tracker algorithm and the global nearest neighbor algorithm in terms of tracking accuracy.

## 1. Introduction

Multi-target tracking (MTT) aims to obtain an estimation of target states from the measurements (localizations, velocities, etc.) received by a sensor in complex scenarios [1,2]. In addition to being widely used in radar, sonar and other surveillance systems, MTT has also been used to obtain the accurate localization of moving targets in sensor networks [3,4]. In conventional radar target tracking systems, measurement data are obtained by a detector with a specific detection probability, and they are then provided to a tracker for target trajectory estimation [2,5]. This radar system simplifies the implementation of the detection and tracking process, thereby easing its computing burden. Nevertheless, this leads to performance degradation, as this simplified structure breaks the internal coupling relationship between the detector and the tracker. 

In order to improve the system performance, a two-way information flow (also known as the integration of detection with target tracking) was developed, in which the tracker informs the detector where to find the target and the detector utilizes this information to better locate the target [6]. In other words, the integration of detection with the target tracking method supplies the detector with feedback information from the tracker, and the feedback has the form of a posterior distribution on the target location. This couple relationship between the detector and the tracker was revealed in [7,8,9,10] by analyzing the Riccati equation iterative operation and track results, which demonstrated that the prior information from the tracker can be used to determine the optimal detection threshold. One advantage of integrating detection with tracking is that the Bayesian detector can use the feedback from the tracker as a priori information for its hypothesis test. Moreover, it is expected to have fewer false alarms and a location-dependent detection threshold—the threshold decreases when it gets close to the estimated target location. This method has been successfully applied to the probabilistic data association (PDA) filter to enhance tracking performance when the detector threshold is adjusted by using the prior information from the PDA tracker [6]. Furthermore, in [11], the idea of the integration of detection with target tracking was extended to multi-target tracking scenarios by integrating the detection with the joint probability data association (JPDA) tracker instead. In this way, the recalculation of joint association probability was enabled, reducing the mutual interference between adjacent targets and, hence, enhancing the performance of the JPDA tracker.

In order to exploit the best of integrating detection with the tracking method in MTT scenarios, alternative data association algorithms besides JPDA are being considered. Among them, multiple hypothesis tracking (MHT) is an optimal multi-target data association algorithm that defers the decision to confirm the tracks. In complex environments with a low detection probability, high density clutter, and/or closely-spaced targets, it has been shown that the performance of MHT is significantly better than JPDA and any other data association algorithms. Normally, MHT algorithms are divided into hypothesis-oriented MHT (HOMHT) [12] and track-oriented MHT (TOMHT) algorithms [13,14,15]. HOMHT maintains the hypothesis structure from scan to scan and exerts the probabilities of global measurement to directly target association hypotheses. HOMHT experiences an exponentially growing global association number and is therefore too complex to be widely adopted. In contrast, TOMHT is favored due to its simplicity in track hypothesis generation. Typically, TOMHT updates the global hypothesis by using newly detected tracks on each scan and utilizes a track tree structure to maintain potential track sets.

The problem of tracking in a dense clutter in which the number of false tracks is large and the quality of the true track is poor has not been adequately addressed in the tracking literature. As the integration structure of detection and tracking can efficiently reduce the number of false tracks, in this paper, we propose a new multiple hypothesis tracking algorthim integrated with detection processing (MHT-IDP) under an efficient TOMHT framework. In our algorithm, the target spatial distribution information from MHT is utilized as a priori knowledge to calculate an adaptive detection threshold. Then, the MHT algorithm estimates trajectories by using the receiving measurements from the adaptive detector. Unlike methods where the detection threshold is fixed, here, the detection threshold is adaptive. As the detection probabilities and clutter density chane with the detection threshold, the adaptive score function and sequential probability ratio test (SPRT) thresholds can be accordingly calculated so as to improve the tracking performance. The effectiveness of the proposed algorithm is verified through the comparison with the standard MHT algorithm and the global nearest neighbor (GNN) algorithm [2] in a dense clutter scenario. 

The rest of this paper is organized as follows. Section 2 briefly introduces the target tracking model and the integration of detection with the target tracking method. In Section 3, the MHT-IDP algorithm is proposed, and its realization process is presented in detail. In particular, we exhibit the calculation of the adaptive detection probability and adaptive clutter density. Section 4 compares the tracking performance of the MHT-IDP algorithm, the MHT method, and the GNN algorithm. Finally, our conclusions are drawn in Section 5.

## 2. Integration of Detection with Target Tracking

The integration of detection with a target tracking algorithm stems from the idea of two-way information flow. The location information of the target is fed back from the tracker to the detector in order to better adjust the detection threshold, obtain more accurate measurements, and, hence, enhance the tracking performance. The detailed derivation of this algorithm can be found in [6].

At scan *k*, the motion model and measurement model of target can be expressed as:

(1) x(k)=Fx(k−1)+v(k)

(2) z(k)=Hx(k)+w(k)

where x(k) and z(k) denote the target state and target measurement at time *k*, respectively; F is the system transition matrix; H is the measurement matrix; v(k) is the system process noise; and w(k) is the measurement noise, which are assumed to be independent zero-mean Gaussian white noises.

At scan *k*, the predicted target state $\hat{x}(k|k-1)$ and predicted measurement $\hat{z}(k|k-1)$ can be obtained by using Kalman filtering. They are then formulated as:

(3) $$\hat{x}(k|k-1)=F\hat{x}(k-1|k-1)$$

(4) $$\hat{z}(k|k-1)=H\hat{x}(k|k-1);$$

and the innovation and the innovation covariance of the true measurement can be computed as:

(5) $$v(k)=z(k)-\hat{z}(k|k-1)$$

(6) $$S(k)=HP(k|k-1)H^{T}+R(k)$$

where P(k|k−1) is the prediction covariance and R(k) is the measurement noise covariance.

In most multi-target tracking systems, gating is a technique for eliminating unlikely measurement-to-track pairings and thereafter reducing the computation of the data association step. The gate is generally described by an ellipsoid region around the predicted measurement position at the next scan:

(7) $$V=\left\{z(k)|d^{2}={\left(z(k)-\hat{z}(k|k-1)\right)}^{T}S{\left(k\right)}^{-1}\left(z(k)-\hat{z}(k|k-1)\right)\leq G\right\}$$

where G is a maximum likelihood gate. Only the measurements that fall in the tracking gate are likely to be associated with the track.

Assume that at scan *k*, $M_{k}$ measurements exist in the gate: $z_{m}(k),m=1,2,\cdots ,M_{k}$. Then, a test of absence or presence of a target at location $z_{m}(k)$ is to be performed. Hypothesis $H_{0}$ indicates that no target exists at location $z_{m}(k)$, whereas hypothesis $H_{1}$ indicates that a target exists at location $z_{m}(k)$. Thus, referring to the derivation of the prior probability in [6], the prior probability of these two hypotheses can be expressed as:

(8) $$P_{H_{1}}\propto \left\{\frac{1}{\sqrt{\left|2\pi S(k)\right|}}\right\}\exp\left\{-\frac{1}{2}v_{m}^{T}S{\left(k\right)}^{-1}v_{m}\right\}$$

(9) PH0∝1V

where $v_{m}(k)=z_{m}(k)-\hat{z}(k|k-1)$ and V denotes the volume of the gate.

Assume that the amplitude of target complies with a Swerling I fluctuation model, and the noise is a Gaussian white noise. The likelihood function can be written as:

(10) $$P\left(a_{m}(k)|H_{0}\right)=\exp\left(-a_{m}(k)\right),\;\mathrm{without}\;\mathrm{target}$$

(11) $$P\left(a_{m}(k)|H_{1}\right)=\frac{1}{1+\rho }\exp\left(-\frac{a_{m}(k)}{1+\rho }\right),\;\mathrm{with}\;\mathrm{target}$$

where $a_{m}(k)$ is the magnitude-square output of a matched filter and ρ is the signal to noise ratio (SNR). According to the Bayesian criterion, the appropriate test can be written as:

(12) $$\frac{P\left(a_{m}(k)|H_{1}\right)}{P\left(a_{m}(k)|H_{0}\right)}\begin{array}{c} \begin{array}{l} H_{1}\\ ≷\\ H_{0} \end{array} \end{array}\frac{P_{H_{0}}\left[c_{10}-c_{00}\right]}{P_{H_{1}}\left[c_{01}-c_{11}\right]},$$

where $c_{ij}$ indicates the cost of judging as i when j is true. Thus, by substituting Equations (8)–(11) into Equation (12), we can obtain the test:

(13) $$a_{m}(k)\begin{array}{c} \begin{array}{l} H_{1}\\ ≷\\ H_{0} \end{array} \end{array}\frac{1+\rho }{2\rho }v_{m}^{T}S{\left(k\right)}^{-1}v_{m}+\eta_{BD}$$

where $\eta_{BD}$ is a parameter independent of $v_{m}$. Therefore, the adaptive detection threshold is:

(14) $$\tau_{m}=\frac{1+\rho }{2\rho }v_{m}^{T}S{\left(k\right)}^{-1}v_{m}+\eta_{BD}.$$

As in Equation (10), the false alarms rate changes with the detection threshold. Let τ denote the detection threshold. The false alarms rate can be computed as $P_{fa}=e^{-\tau }$. Combined with Equation (13), the average false alarm rate in the tracking gate can be calculated as:

(15) $$\begin{array}{ll} P_{fa} & =\int_{V}P(a\geq \tau |v)P_{H_{0}}dv\\ & =\int_{V}e^{-\frac{1+\rho }{2\rho }v^{T}S{\left(k\right)}^{-1}v-\eta_{BD}}\frac{1}{V}dv\\ & \approx \frac{1}{V}e^{-\eta_{BD}}\sqrt{\left|2\pi \frac{\rho }{\rho +1}S(k)\right|} \end{array}$$

Assuming that the average false alarm rate required by the task is a constant denoted as ${\bar{P}}_{fa}$, by substituting it into Equation (15) we can get:

(16) $$\eta_{BD}=\ln\sqrt{\left|2\pi \frac{\rho }{\rho +1}S(k)\right|}-\ln({\bar{P}}_{fa}V).$$

Therefore, the adaptive test can be written as:

(17) $$a_{m}(k)\begin{array}{c} \begin{array}{l} H_{1}\\ ≷\\ H_{0} \end{array} \end{array}\frac{1+\rho }{2\rho }v_{m}^{T}S{\left(k\right)}^{-1}v_{m}+\ln\sqrt{\left|2\pi \frac{\rho }{\rho +1}S(k)\right|}-\ln({\bar{P}}_{fa}V).$$

The measurements which are satisfied with the gating restriction are sent to the tracker, and the tracker makes an measurements-to-track assignment and then updates tracks. For each track, the *SNR* at the current scan *k* is unknown and needs to be estimated from the association results of the previous scan.

## 3. The MHT-IDP Algorithm

**Figure 1:**
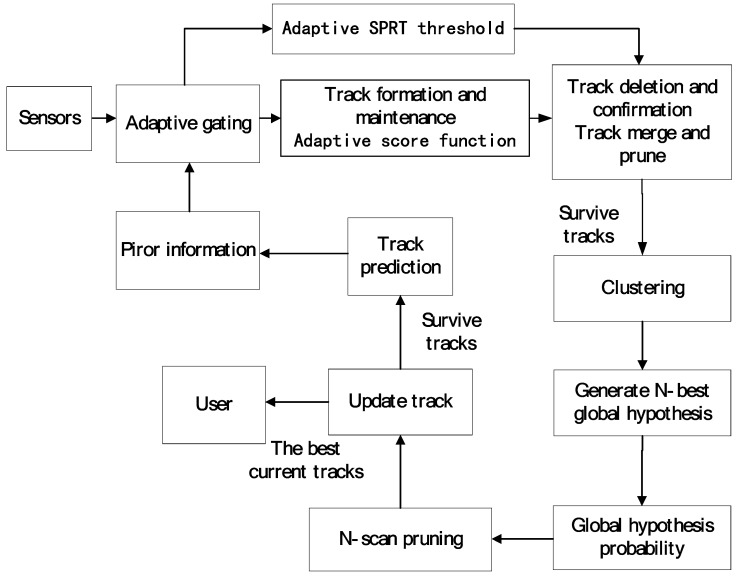
The flowchart of the multiple hypothesis tracking integrated with detection processing (MHT-IDP) algorithm.

In this section, a new multiple hypothesis tracker integrated with detection processing (MHT-IDP) is proposed. The diagram of the MHT-IDP algorithm based on the TOMHT structure [16,17] is shown in Figure 1. The location information is predicted by the MHT algorithm, and we utilize this information to calculate the adaptive threshold. Subsequently, the adaptive score function and the adaptive SPRT thresholds are computed to make the association decision and track judgement. Then, the surviving tracks are clustered, and the global hypothesis of each cluster is calculated. Finally, the tracks that survive after the pruning step are reserved for the next scan. The MHT-IDP algorithm mainly makes improvements on the track score module and the SPRT module. Specifically, the algorithm employs the adaptive detection threshold to calculate the adaptive detection probability and clutter density, and then it obtains a new adaptive scoring function and SPRT threshold. The MHT-IDP algorithm can then make the association decision and adaptively track the judgement according to the adaptive detection threshold. Compared to the commonly-used fixed threshold, adopting an adaptive threshold can effectively improve the accuracy of data association and the quality of the track in complex scenes.

At scan *k*, we assume that *N* tracks exist $T_{t}(k),t=1,2,\cdots ,N$. For track *t*, denote its innovations covariance as $S_{t}(k)$, the volume of tracking gate as $V_{t}$, and *SNR* as $\rho_{t}$.

### 3.1. Adaptive Detection Module

According to the previous section, the adaptive detection threshold that varies with the detection position is shown in Equation (17). In the tracking gate, the detection environment varies with the detection threshold. Therefore, the adaptive detection probability and the adaptive clutter density of the track $T_{t}(k)$ can be calculated by using this adaptive detection threshold.

#### 3.1.1. Detection Probability

The detection probability represents the ratio of the number of targets that exceed the threshold to the existing target number. The detection probability varies with a location-dependent threshold, which means that the measurement in the vicinity of the estimated position exceeding the detection threshold is more likely target than one further away. According to Equation (10), when the threshold is τ, the detection probability can be described as $P_{d}=\exp\left(-\frac{-\ln P_{fa}}{1+\rho }\right)=\exp\left(-\frac{\tau }{1+\rho }\right)$. Thus, in the tracking gate, the average adaptive detection probability can be calculated as:

(18) $$\begin{array}{ll} P_{d} & =\int_{V}P(a\geq \tau |v)P_{H_{1}}dv\\ & =\int_{V}\exp\left[-\frac{1}{\left(\rho +1\right)}\left(\frac{1+\rho }{2\rho }v^{T}S{\left(k\right)}^{-1}v+\eta_{BD}\right)\right]\frac{1}{\sqrt{\left|2\pi S(k)\right|}}\exp\left[\frac{1}{2}v^{T}S{\left(k\right)}^{-1}v\right]dv\\ & ={\left(\frac{\rho }{\rho +1}\right)}^{\frac{n_{z}}{2}}\exp\left[-\frac{\eta_{BD}}{\rho +1}\right] \end{array}$$

where $n_{z}$ is the dimension of the measurements. By substituting Equation (16) into Equation (18), we can obtain the average detection probability of $T_{t}(k)$, which is:

(19) $$P_{d}{}^{t}(k)={\left(\frac{\rho_{t}}{\rho_{t}+1}\right)}^{\frac{n_{z}}{2}}\exp\left[\frac{1}{\rho_{t}+1}\left(-\ln\sqrt{\left|2\pi \frac{\rho_{t}}{1+\rho_{t}}S(k)\right|}+\ln({\bar{P}}_{fa}V_{t})\right)\right].$$

#### 3.1.2. Clutter Density

A Poisson distribution is a natural selection for seeding clutter within the space data cube [2,6,18]. In the tracking process, we generally assume that the spatial distribution of clutter obeys a Poisson distribution. When a fixed-threshold detector is used, the probability mass function (PMF) of the clutter that generates l false alarms in the gate is expressed as:

(20) $$P(l)={\left(\frac{\lambda V}{l!}\right)}^{l}e^{-\lambda V}.$$

Here, λ is the average number of false alarm per unit volume. In the tracking system, λ is commonly referred to as clutter density, and based on the statistical results of the experimental data, the fixed thresholds used in the experiment were set to $\tau_{F}$.

When a detection test is made on the clutter, the amplitude of the detected clutter is retained (or discarded) when it exceeds (or falls below) the threshold τ. Thus, the clutter through the testing threshold still obeys a Poisson point process. It is assumed that the clutter density λ is generated by a Poisson point process with a spatial density of clutter $\tilde{\lambda }$ through a fixed threshold of $\tau_{F}$. Then, it can be written as:

(21) $$\lambda =\tilde{\lambda }e^{-\tau_{F}}$$

Thus, the clutter density of the *m*th measurement in the tracking gate of $T_{t}(k)$ can be expressed as:

(22) $${\overset{⌢}{\lambda }}_{m}^{t}(k)=\tilde{\lambda }e^{-\tau_{m}^{t}}=\tilde{\lambda }e^{\tau_{F}-\tau_{m}^{t}}$$

After substituting Equations (14) and (16) into Equation (22), the new clutter density can be defined as:

(23) $${\overset{⌢}{\lambda }}_{m}^{t}(k)=\frac{\lambda {\bar{P}}_{fa}V_{t}}{\sqrt{\left|2\pi \frac{\rho_{t}}{\rho_{t}+1}S_{t}(k)\right|}}\exp\left(\tau_{F}-\frac{\rho_{t}+1}{2\rho_{t}}v_{m}S_{t}{\left(k\right)}^{-1}v_{m}\right)$$

The clutter density in Equations (22) and (23) is decided by the test threshold, which varies with the measurement location.

### 3.2. Adaptive Score Function

The probability of the track is evaluated by the track score, which includes all aspects of the data association problem, and each track corresponds to one track score. In the traditional score function, the environment parameters and the detection probability are fixed. Thus, when the detection environment changes, the track score error grows, and the accuracy of the data association deteriorates accordingly. In order to improve the accuracy of data association, this section utilizes the adaptive detection probability and clutter density to calculate the score increment. The detail derivation of the adaptive score increment can be described as follows.

The track score can be expressed by the log likelihood ratio of the association hypothesis [2]. At scan *k*, a recursive form of the track score can be written as:

(24) L(k)=L(k−1)+ΔL(k)

We assume that there exist $M_{k}^{t}$ measurements $z_{m}(k),m=1,2,\cdots ,M_{k}^{t}$ in the tracking gate of $T_{t}(k-1)$. $z_{0}$ represents the fact that no detection has occurred. Then, the increment of the corresponding track score for each likely measurement-track pair can be expressed as:

(25) $$\Delta L_{m}^{t}(k)=\left\{ \begin{array}{ll} \ln(1-P_{d}), & m=0\\ \ln\left[\frac{P\left\{z_{m}(k)|T_{t}(k-1)\right\}P_{d}}{\lambda +\lambda_{v}}\right], & m\neq 0 \end{array}\right.$$

where $P_{d}$ is the detection probability and if no update on scan *k*, the increment of score is $\ln(1-P_{d})$, $\lambda_{v}$ indicates the density of new target, λ represents the density of clutter, and $P\left\{z_{m}(k)|T_{t}(k-1)\right\}=\frac{1}{{\left(2\pi \right)}^{n_{z}/2}{\left|S_{t}(k)\right|}^{1/2}}\exp\left\{-\frac{1}{2}\left(v_{m}S_{t}{\left(k\right)}^{-1}v_{m}\right)\right\}$ represents the likelihood ratio of associating the track $T_{t}(k-1)$ with the measurements $z_{m}(k)$ at scan *k*.

From Section 3.1.1, we know that the detection probability $P_{d}^{t}$ and the clutter density ${\overset{⌢}{\lambda }}_{m}^{t}$ vary with the detection threshold. After substituting Equations (19) and (23) into Equation (25), the adaptive increment of score function can be represented as:

(26) $$\Delta L_{m}^{t}(k)=\left\{ \begin{array}{ll} \ln(1-P_{d}^{t}), & \;m=0\\ \ln\left[\frac{P\left\{z_{m}(k)|T_{t}(k-1)\right\}P_{d}^{t}}{{\overset{⌢}{\lambda }}_{m}^{t}+\lambda_{v}}\right], & m\neq 0 \end{array}\right..$$

### 3.3. Adaptive SPRT Threshold

We make use of the SPRT to test the track score, and its value determines the actions of confirming the track, deleting the track, or continuing to test the track. The traditional SPRT uses fixed upper and lower detection thresholds to test with respect to the track score. When the detection scenario changes, track judgement accuracy decreases, and the number of intermittent tracks and false tracks increases. Therefore, in complex scenarios, we use the adaptive detection threshold instead to calculate the adaptive false track confirmation probability at each scan in order to enhance the quality of the track judgment and generation. Thereafter, an adaptive SPRT threshold can be obtained.

**Figure 2:**
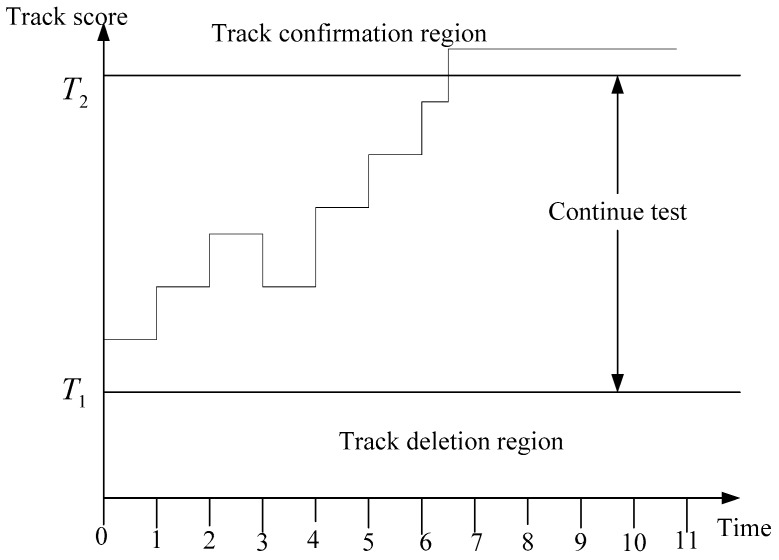
The score function method as an application of the sequential probability ratio test (SPRT).

The logic of the SPRT [2,19,20,21] to test track is shown in Figure 2:

The standard SPRT test thresholds are defined as:

(27) $$\left\{ \begin{array}{l} T_{2}=\ln\left[\frac{1-\beta }{\alpha }\right]\\ T_{1}=\ln\left[\frac{\beta }{1-\alpha }\right] \end{array}\right.$$

where α represents false track confirmation probability and β represents the true track deletion probability.

The false track confirmation probability is defined from the requirements on false track initiation in the system [2]. For instance, assuming that the system produces $N_{fa}$ false alarms per second and it permits $N_{fc}$ false alarms confirmation per hour, the probability that any false alarm generates a false track is:

(28) $$\alpha =\frac{N_{fc}}{3600N_{fa}}$$

In the MHT-IDP algorithm, by dynamically adjusting the test threshold parameter $\eta_{BD}$, the false alarm rate in each tracking gate can be kept as a constant ${\bar{P}}_{fa}$. Thus, at scan *k,* the changing false alarm number in the tracking gate of track $T_{t}(k)$ can be expressed as:

(29) $$N_{fa}^{t}(k)={\bar{P}}_{fa}M_{k}^{t}.$$

The number of false alarms which are generated by the system is:

(30) $$N_{fa}(k)=\sum_{t=1}^{N}N_{fa}^{t}(k)=\sum_{t=1}^{N}{\bar{P}}_{fa}M_{k}^{t}$$

where N is the number of existing tracks at scan *k*. After substituting Equation (30) into Equation (28), the false track confirmation probability can be calculated as:

(31) $$\alpha (k)=\frac{N_{fc}}{3600\sum_{t=1}^{N}{\bar{P}}_{fa}M_{k}^{t}}.$$

Since the influence of β on the track confirmation threshold is very small, the choice of β is unimportant, and we can thus limit β≤0.1 for the calculation of $T_{2}$. Then, the low-score track deletion rule is defined according to the track maintenance capability of the system. 

In the MHT-IDP algorithm, the newly adaptive SPRT threshold can be written as:

(32) $$\left\{ \begin{array}{l} T_{2}\left[\alpha (k),\beta \right]=\ln\left[\frac{1-\beta }{\alpha (k)}\right]\\ T_{1}\left[\alpha (k),\beta \right]=\ln\left[\frac{\beta }{1-\alpha (k)}\right] \end{array}\right..$$

Thus, at scan *k*, the logic of the adaptive SPRT to process the measurements is:

(33) $$L\left(T_{t}(k)\right)\left\{ \begin{array}{l} \leq T_{2}\left[\alpha (k),\beta \right],\;\mathrm{delete}\\ \geq T_{1}\left[\alpha (k),\beta \right],\;\mathrm{confirm}\\ \mathrm{otherwise},\;\mathrm{continue}\;\mathrm{track} \end{array}\right.$$

The module of the adaptive SPRT manages the tracks by deleting the low-score tracks, which reduces unnecessary calculation. We cluster the tracks, form the global hypothesizes, and then deliver the tracks that the survive pruning step to the next scan. The MHT-IDP pseudo-code is presented in Algorithm 1.

**Algorithm 1** Pseudo-code of the MHT-IDP algorithm.**Input:** the measurement data z(*k*). **Output:** the best tracks $T_{t}(k),t=1,2,\cdots ,N.$ 1: Set *k*=1. 2: **for**
*i*=1→length (z(*k*)) 3:  calculate the adaptive threshold *τ_i_*(*k*) with (14) 4:  **if** amplitude *a_i_*(*k*) > *τ_i_*(*k*) 5:    calculate the adaptive detection probability $P_{d}^{i}(k)$ with (19) and calculate the clutter density and ${\overset{⏜}{\lambda }}_{m}^{i}(k)$ with (22). 6:    calculate the adaptive score function $\Delta L_{m}^{i}(k)$ with (26) and acquire the changing alarm number $N_{fa}^{i}(k)$ with (29). 7:  **end if** 8: **end for** 9: calculate the track score *L_t_*(*k*), *t* = 1,2,...,*N*. with (24). 10: calculate the adaptive SPRT threshold with (31)and (32) 11: **for**
*t* = 1→N 12:  **if**
*L_t_*(*k*) > *T_1_*(*k*), confirm the track, **end if** 13:  **else if**
*T_2_*(*k*) ≤ *L_t_*(*k*) ≤ *T_1_*(*k*), continue to test track, **end if** 14:  **else if**
*L_t_*(*k*) < *T_2_*(*k*), delete the track, **end if** 15: **end for** 16: cluster the tracks, form the global hypothesizes and N-best pruning the tracks.17: Set *k* = *k*+1, return the predicted data $\hat{z}(k+1)$ to step 2.

## 4. Experimental Results

In this section, we evaluate the MHT-IDP algorithm in the dense clutter simulation scenarios by several performance metrics. Comparisons were made with the standard MHT algorithm and the GNN algorithm in order to demonstrate the MHT-IDP algorithm’s superiority in tracking performance.

### 4.1. Simulation Scenairo. 

**Figure 3:**
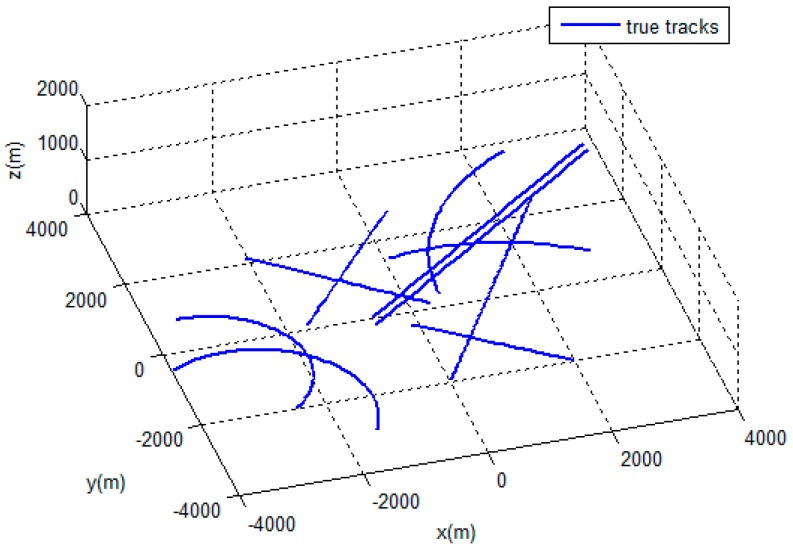
Target trajectories.

**Figure 4:**
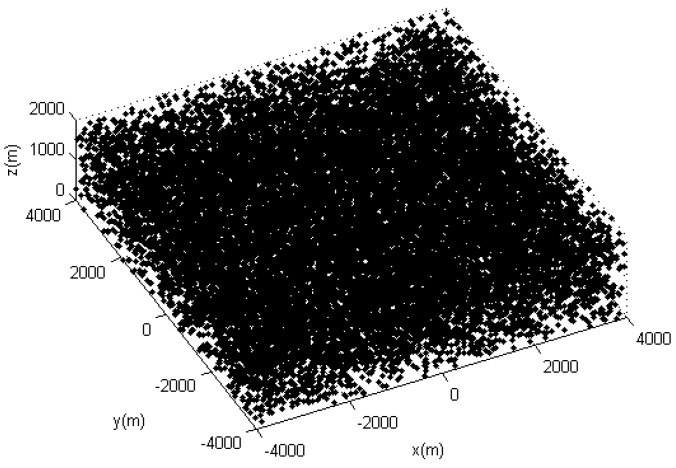
The real measurements with clutter.

In the simulation scenario, we had 10 motion targets distributed in a space of [−4000, 4000 *m*] × [−4000, 4000 *m*] × [0, 1000 *m*]. We assumed a sampling interval T=1s. The measurements covered 100 scans. Figure 3 shows the trajectories of the target, and Figure 4 shows the target measurements with the dense clutter.

The state space model of the filter was a constant velocity (CV) model. Targets moved in a 3D surveillance area. At scan *k*, the target state was $x_{k}={\left[p_{x,k},{\dot{p}}_{x,k},p_{y,k},{\dot{p}}_{y,k},p_{z,k},{\dot{p}}_{z,k}\right]}^{T}$ which included the information of velocity and location in the *X–Y–Z* coordinate system. The state transition matrix F, the measurement matrix H, the covariance matrix of process noise Q, and the covariance matrix of measurement noise R were defined as follows

$$F=\left[\begin{array}{ccc} F^{1} & 0 & 0\\ 0 & F^{1} & 0\\ 0 & 0 & F^{1} \end{array}\right],F^{1}=\left[\begin{array}{cc} 1 & T\\ 0 & 1 \end{array}\right];H=\left[\begin{array}{cccccc} 1 & 0 & 0 & 0 & 0 & 0\\ 0 & 0 & 1 & 0 & 0 & 0\\ 0 & 0 & 0 & 0 & 1 & 0 \end{array}\right]$$

$$Q=\left[\begin{array}{ccc} Q^{1} & 0 & 0\\ 0 & Q^{1} & 0\\ 0 & 0 & Q^{1} \end{array}\right]\delta_{v}^{2},Q^{1}=\left[\begin{array}{c} \frac{1}{2}T^{2}\\ T \end{array}\right];R=\left[\begin{array}{ccc} 1 & 0 & 0\\ 0 & 1 & 0\\ 0 & 0 & 1 \end{array}\right]\delta_{\varepsilon }^{2}$$

where the standard deviation of process noise was $\delta_{v}=50m^{3}$ and the standard deviation of measurement noise was $\delta_{\varepsilon }=50m^{3}$. The density of the new target was $\lambda_{v}=1\times 10^{-11}/m^{3}$. The density of clutter was $\lambda =2\times 10^{-4}/m^{3}$, the average false alarm rate was ${\bar{P}}_{fa}=10^{-6}$, and the true track deletion probability was $\beta =10^{-3}$. To verify the performance of the proposed algorithm, we compared the proposed algorithm with the MHT algorithm and the GNN algorithm under 100 Monte Carlo simulations.

### 4.2. Results and Evaluation.

In this part, we compare the performance of three algorithms with respect to the miscorrelation rate, the correct correlation rate, the average number of false tracks, the average track maintenance time, and the optimal sub pattern assignment distance. The detailed description of these metrics was introduced in [22,23,24,25,26,27,28].

(a) The correct correlation rate of true tracks ($R_{CC}$) is defined as the ratio of the total number of observations correctly associated with true tracks $N_{CC}$ to the number of the observations that originate from targets $N_{OT}$. The correct correlation rate of the true tracks is one of the metrics to evaluate the quality of data correlation. It can be expressed as:

(34) $$R_{CC}=\frac{N_{CC}}{N_{OT}}.$$

(b) The miscorrelation rate of true tracks ($R_{MC}$) is also used for evaluating the data association quality. It is defined as the ratio of the average number of miscorrelation $N_{MC}$ over the average track life $T_{AT}$, and it can be calculated as:

(35) $$R_{MC}=\frac{N_{MC}}{T_{AT}}$$

(c) The average number of false track ($\bar{N_{ft}}$) is defined as the average number of false tracks over the Monte Carlo simulations. When assuming that the *k*th Monte Carlo simulation produces $N_{ft}(k)$ false tracks and the total number of Monte Carlo simulation is $N_{M}$, then the average number of false track is:

(36) $$\bar{N_{ft}}=\frac{\sum_{N=1}^{N_{M}}N_{ft}(k)}{N_{M}}.$$

(d) The average track maintenance time ($T_{Am}$) metric is defined to evaluate the performance of track maintenance in each scan. If we suppose at scan *k* that the duration of track m is $T_{dt}(m)$ and the total number of tracks is $N_{N}$, then the average track maintenance time can be calculated as:

(37) $$T_{Am}(k)=\frac{\sum_{m=1}^{N_{N}}T_{dt}(m)}{N_{N}}$$

(e) The average processing time for per scan ($T_{H}$)metric is defined to evaluate the tracker computational complexity in seconds.

(f) The optimal sub pattern assignment (OSPA) distance is defined to measure the accuracy of cardinality and state estimation. The OSPA distance between the set of the target real state $X=\left\{x_{1},\cdots ,x_{n}\right\}$ and the set of the estimated state $Y=\left\{{\hat{y}}_{1},\cdots ,{\hat{y}}_{m}\right\}$ is calculated by

(38) $${\bar{d}}_{p}^{\left(c\right)}(X,Y)=\left\{ \begin{array}{l} {\left\{\frac{1}{n}\left[\underset{\pi \in \Pi_{n}}{\min}\sum_{i=1}^{m}d^{\left(c\right)}{\left(x_{i},{\hat{y}}_{\pi (i)}\right)}^{p}+c^{p}(n-m)\right]\right\}}^{1/p},\;n\geq m\\ {\bar{d}}_{p}^{\left(c\right)}(Y,X),\;\;\;\;\;\;\;\;\;\;\;\;\;\;\;\;\;\;\;\;\;\;\;\;\;\;\;\;\;\;\;\;\;\;\;\;\;\;\;\;\;\;\;\;\;\;\;\;\;\;\;\;\;\;\;\;\;\;\;\;\;\;n<m \end{array}\right.$$

where $\Pi_{n}$ represents the set of all possible permutations of {1,2,⋯,n}, and $d^{\left(c\right)}(x,y)=\min(c,d(x,y))$ is the truncated Euclidean distance between the vectors x and y. In our simulations, the cut-off distance c was set to 200, and the order parameter p was set to 2. 

**Figure 5:**
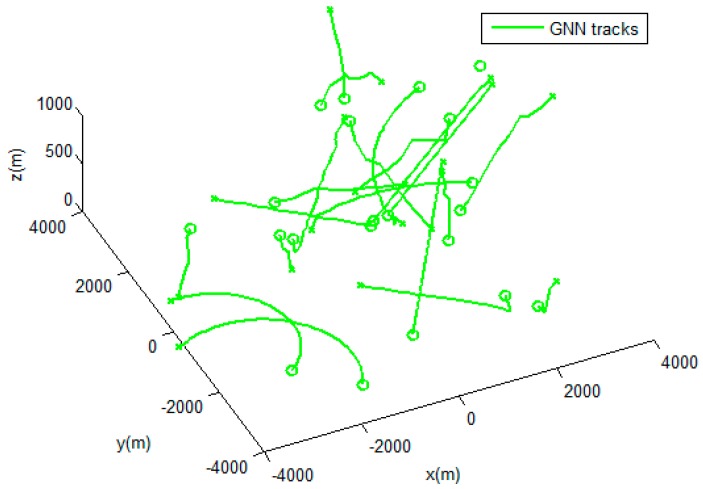
The estimated trajectories of the global nearest neighbor (GNN) algorithm.

**Figure 6:**
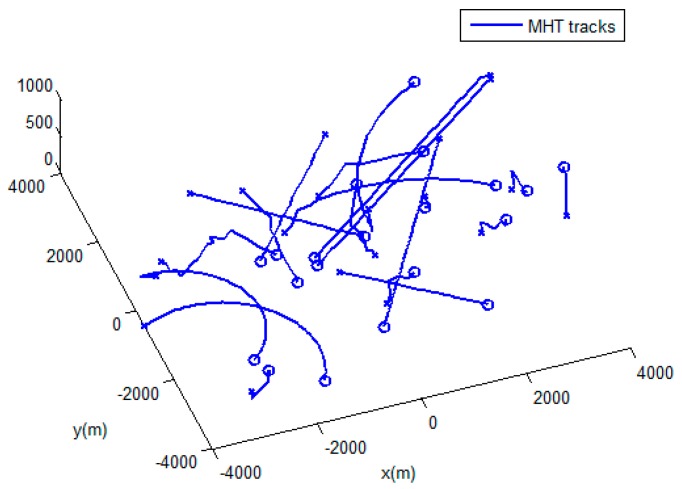
The estimated trajectories of the MHT algorithm.

**Figure 7:**
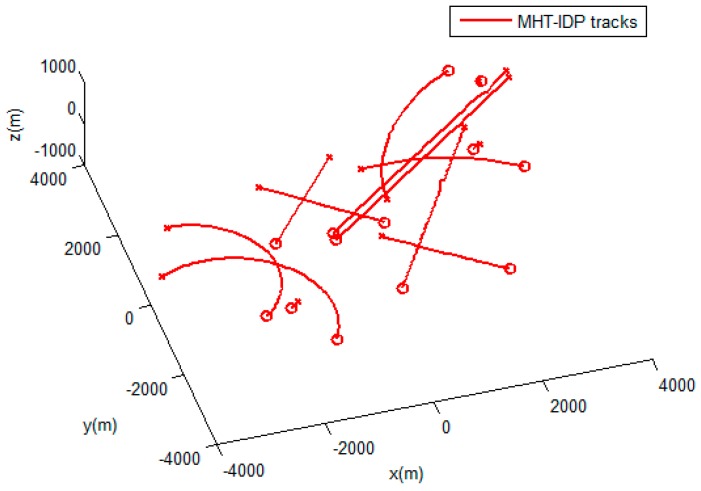
The estimated trajectories of the MHT-IDP algorithm.

**Figure 8:**
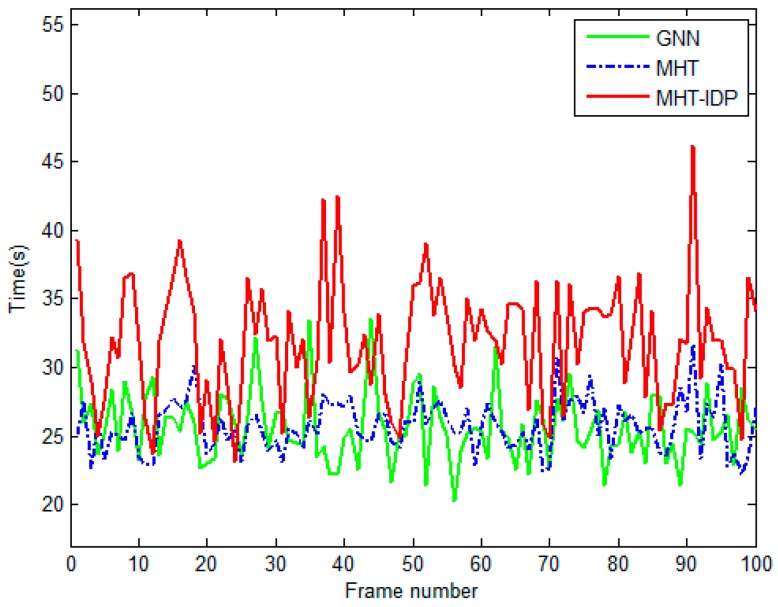
The average track maintenance time of three algorithms.

**Figure 9:**
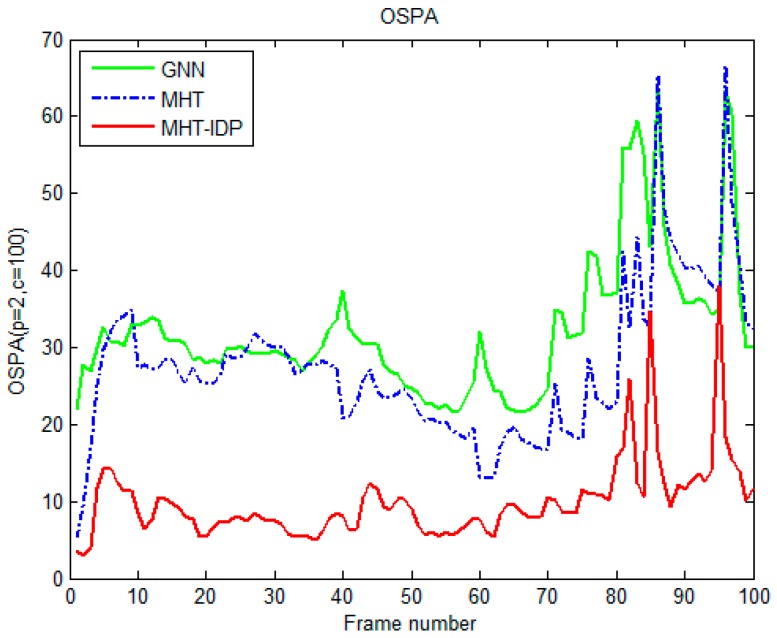
The optimal sub pattern assignment (OSPA) distance of three algorithms.

**Figure 10:**
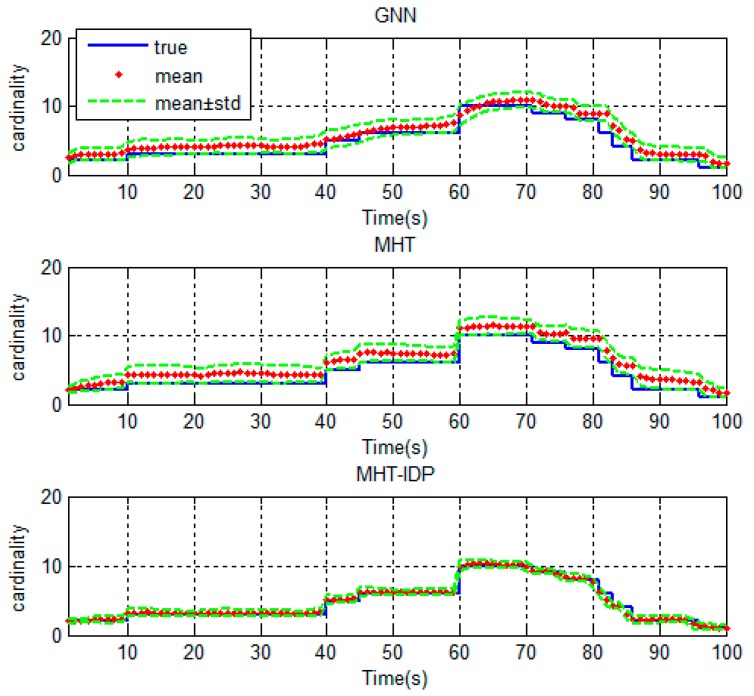
The cardinality estimation of three algorithms.

**Figure 11:**
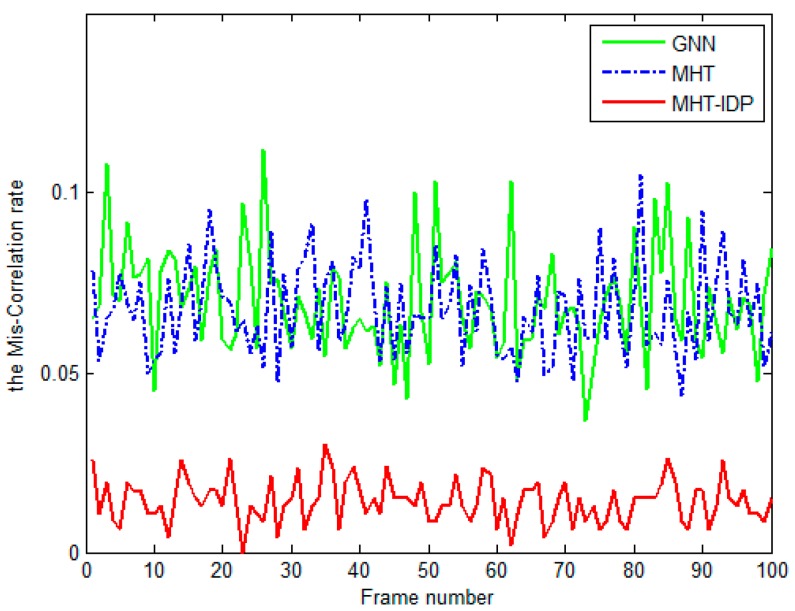
The miscorrelation rate of true tracks of three algorithms.

The performances of the MHT-IDP algorithm, the MHT algorithm, and the GNN algorithm are shown in Table 1 and Figure 5, Figure 6, Figure 7, Figure 8, Figure 9, Figure 10 and Figure 11. It can be clearly seen that, compared to the conventional GNN algorithm and the MHT algorithm, the MHT-IDP algorithm had fewer false tracks, less clutter, and less mutual interference; thus, it presented a better tracking quality.

Table 1 illustrates the tracking performance of the three algorithms under several metrics. Compared with the MHT algorithm and the GNN algorithm, the MHT-IDP algorithm had a lower miscorrelation rate of true tracks $R_{MC}$ and a higher correct correlation rate of true tracks $R_{CC}$. Thus, the MHT-IDP algorithm had a better accuracy of data association. Furthermore, the MHT-IDP algorithm had a smaller average number of false tracks $\bar{N_{ft}}$. This means that the MHT-IDP algorithm could effectively reduce the number of false tracks. Moreover, the MHT-IDP algorithm had a longer average processing time for per scan $T_{H}$ than the MHT algorithm and the GNN algorithm, which means that the MHT- IDP algorithm obtained better tracking effect at the expense of more computation time.

Figure 8 shows the comparison results of the average track maintenance time of the three algorithms. The average track maintenance times of the GNN algorithm, the MHT algorithm, and the MHT-IDP algorithm were 28.92, 27.12, and 34.91 s, respectively. According to Figure 8, the MHT-IDP algorithm improved the track continuity, reduced the interference of false track, and effectively increased the track maintenance time.

Figure 9 and Figure 10 show the OSPA distance and the cardinality estimation of three algorithms. It can be seen that the MHT-IDP algorithm had a lower average OSPA distance. Moreover, the mean value of cardinality estimation was closer to the actual target number, and the covariance of cardinality estimation was smaller. These simulation results prove that the proposed algorithm can acquire better estimation performance.

Figure 11 shows the true tracks miscorrelation rate of three algorithms. The means of the miscorrelation rate of true tracks of the GNN algorithm, the MHT algorithm, and the MHT-IDP algorithm were 0.0697, 0.0676 and 0.0142, respectively. According to Figure 11, we can see that the MHT-IDP algorithm effectively reduced the miscorrelation rate of true tracks.

## 5. Conclusions

In this paper, we proposed a new multiple hypothesis tracker, MHT-IDP, in which tracking is integrated with the detection process. This algorithm obtains adaptive thresholds by using the coupling relationship between the detector and the tracker, and it calculates the corresponding adaptive score function and adaptive SPRT thresholds in order to improve the data association and track management performance. To illustrate the effectiveness of the proposed algorithm in both data association and track management, we compared the MHT-IDP algorithm with the standard MHT algorithm and the GNN algorithm in a dense-clutter scenario. We found that the proposed MHT-IDP algorithm can efficiently suppress the false alarm tracks and obtains a better tracking effect at the expense of more computation time. Specifically, it has a higher correct correlation rate of true tracks, a smaller average number of false tracks, and a lower miscorrelation rate of true tracks, as well as a lower OSPA distance and a more accurate cardinality estimation.

## Figures and Tables

**Table 1:** Simulation results of three algorithms.

Algorithm	$R_{CC}$	$\bar{N_{ft}}$	$T_{H}$
GNN	0.901	10	0.283s
MHT	0.924	11	0.309s
MHT-IDP	0.982	3	0.486s
